# Virtual Versus In-Person Intensive Outpatient Treatment for Eating Disorders During the COVID-19 Pandemic in United States–Based Treatment Facilities: Naturalistic Study

**DOI:** 10.2196/66465

**Published:** 2025-05-02

**Authors:** Dan V Blalock, Philip S Mehler, Deborah M Michel, Alan Duffy, Daniel Le Grange, Anne M O'Melia, Renee D Rienecke

**Affiliations:** 1 Center of Innovation to Accelerate Discovery and Practice Transformation Durham Veterans Affairs Medical Center Durham, NC United States; 2 Department of Psychiatry and Behavioral Sciences School of Medicine Duke University Durham, NC United States; 3 Eating Recovery Center/Pathlight Mood and Anxiety Centers Denver, CO United States; 4 Acute Center for Eating Disorders at Denver Health Denver, CO United States; 5 School of Medicine University of Colorado Denver, CO United States; 6 Department of Psychiatry and Behavioral Sciences School of Medicine Tulane University New Orleans United States; 7 Department of Psychiatry and Behavioral Sciences University of California San Francisco, CA United States; 8 Department of Psychiatry & Behavioral Neuroscience The University of Chicago (Emeritus) Chicago, IL United States; 9 Department of Psychiatry School of Medicine University of Utah Salt Lake City United States; 10 Eating Recovery Center/Pathlight Mood & Anxiety Center Denver, CO United States; 11 Department of Psychiatry and Behavioral Sciences Northwestern University Chicago, IL United States

**Keywords:** eating disorders, virtual care, intensive outpatient, COVID-19, higher level of care, depression, suicidal ideation, treatment, access, naturalistic

## Abstract

**Background:**

While virtual therapy has proven effective in treating eating disorders (EDs), little work has examined virtual therapy at higher levels of care, which are treatment options providing more support than weekly outpatient therapy including intensive outpatient (IOP) treatment.

**Objective:**

This study aimed to add to the limited research on in-person versus virtual treatment at a higher level of care by comparing treatment outcomes between an in-person IOP and a virtual IOP (VIOP) for patients with EDs. We hypothesized that there would be no differences in improvements between VIOP and in-person IOP groups.

**Methods:**

This study has a nonrandomized multiple cohort design. Patients with EDs receiving treatment who completed both admission and discharge questionnaires in VIOP treatment (n=231) and in-person IOP treatment (n=39) between 2021 and mid-2022 within a large ED health care system in the United States were included. The Eating Disorder Examination–Questionnaire (EDE-Q) was used to measure ED symptoms. The Patient Health Questionnaire-9 (PHQ-9) was used to measure depression, and item 9 of the PHQ-9 was used to measure suicidal ideation. Welch *t* tests on admission, discharge, and raw change scores were conducted. Logistic regressions were conducted predicting treatment program (reference group VIOP vs in-person IOP) from the residualized change in each outcome and were adjusted for all significantly different factors between groups.

**Results:**

VIOP patients were significantly older (mean 28.03, SD 11.09) than in-person IOP patients (mean 19.51, SD 6.98) and displayed significantly different numbers of ED diagnoses and more comorbid psychiatric diagnoses (VIOP: mean 1.23, SD 1.12; in-person IOP: mean 0.33, SD 0.84) but no differences in race (VIOP: 175/231, 75.6% White; in-person IOP: 30/39, 76.9% White), gender (VIOP: 196/231, 84.8% female; in-person IOP: 35/39, 89.7% female), or length of stay (VIOP: mean 58.84, SD 26.69; in-person IOP: mean 57.33, SD 19.67). When compared to in-person IOP patients, controlling for age, diagnosis, number of comorbid diagnoses, and admission scores, VIOP patients did not exhibit significantly different improvements in ED symptom scores (EDE-Q Global: b=0.01, SE 0.18, *t*=0.04, odds ratio [OR] 1.01, 95% CI 0.71-1.43; *P*=.97). However, VIOP patients exhibited significantly greater improvements in depression scores (PHQ-9: b=–0.14, SE 0.05, *t*_230_=–2.85, OR 0.87, 95% CI 0.79-0.96; *P*=.004) and the PHQ-9 suicidal ideation item (PHQ-9 item 9: b=–0.72, SE 0.34, *t*_230_=–2.13, OR 0.49, 95% CI 0.25-0.93; *P*=.03).

**Conclusions:**

ED outcomes were similar for VIOP and in-person IOP patients. Contrary to our hypotheses, depression and suicidal ideation outcomes improved more for VIOP patients than for in-person IOP patients. Furthermore, treatment access for non-White and older adults does not appear descriptively worse for VIOP treatment compared to in-person IOP treatment, though these trends should be further explored. VIOP treatment may improve treatment access in an equitable fashion without reducing treatment quality.

## Introduction

The COVID-19 pandemic exacerbated almost every mental health disorder, including anxiety, depression [1], and eating disorders (EDs) [2]. EDs are associated with high rates of morbidity and mortality [3], and poor quality of life [4]. Schlegl et al [2] found that while 70% of patients with EDs reported an increase in symptoms after the onset of COVID-19, access to in-person therapy decreased by 37% at the same time. Only 26% of patients surveyed used videoconferencing for therapy. In addition, and contrary to the promise of more access, telehealth modalities have been shown to sometimes increase disparities due to race and age [5].

Studies have found that virtual therapy can be effective in treating EDs [6,7], but an important and less-studied consideration is whether virtual treatment is as effective as in-person treatment. A randomized controlled trial of 128 adults conducted before the COVID-19 pandemic found that outpatient cognitive-behavioral therapy for bulimia nervosa was comparable whether delivered face-to-face or via telehealth [8]. A more recent study of 49 adults found similar results; virtual therapy resulted in comparable improvements in ED symptoms, weight gain, and patient satisfaction when compared to in-person outpatient treatment for patients with different EDs [9]. Even fewer studies have examined virtual treatment in the context of higher levels of care, such as intensive outpatient (IOP) treatment. IOPs can provide a valuable treatment option either for those who need more support than can be provided with weekly outpatient therapy, or for those who are stepping down from a higher level of care, such as a partial hospitalization program (PHP) or residential treatment. The opportunity to continue the recovery process in the less structured treatment environment of the IOP can aid in generalizing skills learned in the higher level of PHP. While there are some variations in the structure and implementation of IOPs (eg, time-limited vs not and closed IOPs vs open, rolling IOPs), the majority of IOPs focusing on EDs maintain a fairly similar structure [10].

A virtual intensive outpatient program (VIOP) for 57 adult patients with EDs was found to be both feasible and acceptable and resulted in clinically meaningful improvements in mood and ED symptoms [11]. Only 4 studies have compared a virtual treatment to an in-person higher level of care treatment for EDs [12-15]. In these studies, 3 of which included adults and one that included adults and adolescents, patients who participated in an in-person IOP or PHP before the onset of the COVID-19 pandemic were compared to patients participating in VIOP or VPHP during the COVID-19 pandemic, with cell sizes ranging from 33 to 70. Significant improvements were found in ED symptoms, depression, perfectionism, and BMI, with no differences between treatment conditions. These findings are important, as telehealth services are not only critical during the pandemic but may also circumvent some of the most commonly cited barriers to seeking treatment, including stigma (eg, it may be easier to see a therapist via telehealth rather than presenting to an ED clinic in person), practical barriers such as transportation and time [16], and lack of treatment services within geographic proximity [17].

The purpose of this study was to compare treatment outcomes and satisfaction between an in-person IOP and a VIOP for patients with EDs within the same health care system. Based on research so far, albeit limited, it was hypothesized that there would be significant improvements in both treatment conditions, with no differences between treatment modalities. We had no a priori hypotheses about treatment satisfaction between the groups.

## Methods

### Participants and Procedure

Participants included 231 patients who received VIOP treatment between January 2021 and mid-2022 and 39 patients who received onsite in-person IOP treatment between mid-2021 and mid-2022. Of all patients, 37 were between the ages of 11 and 17 years (15 receiving VIOP treatment and 22 receiving in-person IOP treatment). Participants received care within a large US ED private health care system, which offers specialized intensive care for those with a variety of EDs including, but not limited to, anorexia nervosa, bulimia nervosa, and binge eating disorder. Between January 2021 and mid-2021, COVID-19–related restrictions were in place preventing in-person IOP treatment, and therefore all patients during this time frame participated in VIOP treatment. Between mid-2021 and mid-2022, those COVID-19–related restrictions were lifted, and patients were able to attend either VIOP or in-person IOP based on a combination of clinical appropriateness and patient preference. Clinical appropriateness for all levels of care, including VIOP, is described in the following paragraph.

All patients were seeking intensive treatment for EDs beyond what is offered in standard outpatient settings, and no active recruitment was conducted for this study. No specific referral was required, though many patients were referred by outpatient providers, family members, or former patients. The level of care recommendation was determined upon assessment via clinical interview using an adaptation of *The American Psychiatric Association Practice Guideline for the Treatment of Eating Disorders, Third Edition* [18], which delineates appropriate criteria for various levels of care. The modified guideline includes domains of illness severity as outlined by The Level of Care Utilization System (LOCUS) for Psychiatric and Addiction Services, Adult Version 20 [19], and the Child and Adolescent Level of Care Utilization System (CALOCUS), Child and Adolescent Version 20 [20], which are instruments to guide consistent assessment and level of care recommendations [21]. Thus, patients with moderate eating disorder symptom severity (Table S1 in Multimedia Appendix 1) were admitted to an IOP level of care. Moderate ED symptom severity with respect to binge eating was defined as 4-7 binge eating episodes per week. It was also defined as 4-7 purging episodes per week for compensatory purging behaviors (eg, self-induced vomiting, laxatives, and diuretics). For patients engaging in restricting behaviors, their meals require external structure and supervision. It is noted that for binge eating and compensatory behaviors, symptom frequency could be slightly less depending on the overall symptom presentation in domains of illness severity as measured by the LOCUS and CALOCUS. Other areas of evaluation are outlined in Table S1 in Multimedia Appendix 1. The discharge date was based on a combination of patient progress and patient preference.

For assessment, adults, or minors alongside parents or guardians, completed clinical interviews with master’s level licensed mental health therapists or social workers to determine preliminary diagnosis (or diagnoses) and appropriate treatment level using the aforementioned guidelines. The assessment process for VIOP was the same as that for in-person IOP. Care recommendations were not based on private health insurance status. However, before admission, insurance authorization was obtained for all patients based on the clinical interview assessment. As a US-based health care system with US-based insurance providers, this reflects affirmation from insurance company reviewers with level of care recommendations based on reported eating disorder symptom severity and the other domains of functioning as outlined in the paper. It is possible, and even likely, that health care systems in other countries or cultural contexts may not necessarily adopt the same level of treatment for all patients reported in this sample. Self-report measures evaluating symptomatology were obtained after the initial admission appointment and were not used in level-of-care recommendations. These measures were completed through patients’ internet-based secure patient portal.

All patients admitted directly to these programs with no previous higher level of care treatment (eg, residential or PHP program). All patients had insurance coverage, and patients in VIOP qualifying for financial need received a food allowance for groceries to offset the cost of meals. Meals were provided onsite with in-person IOP. There were no cost differences based on the modality of care (ie, virtual vs onsite).

### Measures

The Eating Disorder Examination – Questionnaire (EDE-Q) is a 28-item self-report questionnaire assessing the cognitive and behavioral psychopathology of EDs [22-24]. The measure has four subscales: restraint, eating concern, shape concern, weight concern, and a global score. It has been found to have acceptable construct validity, internal consistency, and discriminant validity [25].

The Patient Health Questionnaire-9 (PHQ-9) is a self-report questionnaire consisting of 9 items assessing depressive symptoms, and one item assessing functional impairment. Items are measured on a 4-point scale from “0” (not at all) to “3” (nearly every day) [26]. Scores from 1-4 indicate minimal depression, 5-9 indicate mild depression, 10-14 indicate moderate depression, 15-19 indicate moderately severe depression, and 20-27 indicate severe depression. Item 9 of the PHQ-9 assesses suicidal ideation by asking if the respondent has had “thoughts that you would be better off dead, or of hurting yourself.” The PHQ-9 has been found to have good criterion validity, construct validity, and excellent internal reliability [26].

### Patient Satisfaction

The question, “On a scale of 0-10 where 10 is ‘most likely’ and 0 is ‘least likely’, how likely are you to recommend the [treatment facility] to a friend or other in need?” was used to calculate a Net Promoter Score (NPS) [27]. To establish an NPS, participants are placed into 3 categories: “Promoters” answer the question with scores of 9 or 10, “Passives” answer the question with scores of 7 or 8, and “Detractors” answer it with scores between 0 and 6. The percentage of Detractors is subtracted from the percentage of Promoters to calculate an NPS. NPS scores can range from –100 to 100. According to a recent Retently study, the average NPS for health care providers is 38 [28].

### VIOP Treatment

VIOP participants received treatment that included 3, 3-hour group sessions weekly, 1 hour of individual or family therapy weekly, one weekly 30-minute appointment with a registered dietitian, and regular remote medical monitoring consisting of blood pressure, heart rate, and weight. Virtual care was delivered via the RingCentral Meetings platform, typically on patients’ own tablet or computer devices unless one was provided for them based on financial need. Patients joined one of several groups that occurred at a consistent schedule each week (eg, Monday, Tuesday, and Thursday from 5 to 8 PM), with no more than 11 patients in each group, which varied slightly based on actual attendance.

All group sessions were conducted with either a licensed mental health professional or a registered dietitian. Weights were obtained using numberless scales that were connected to the dietitian’s account. The first hour of the group was a skills-acquisition group based on dialectical behavior therapy [29], cognitive behavioral therapy [23], or acceptance and commitment therapy [30]. The second hour consisted of a supported meal therapy group in which meals were monitored via video and the mobile app Recovery Record was used to record food and beverage intake for dietitian review and feedback [31]. The final hour consisted of process-oriented group therapy twice a week, with the third day using another skills acquisition group. Therapy with a licensed psychotherapist consisted of individual psychotherapy or family therapy and was also based on dialectical behavior therapy, cognitive behavioral therapy, and acceptance and commitment therapy approaches. Recovery Record was also used by patients to record all food and beverage intake throughout the week, which was reviewed by both the dietitian and therapist for evaluation, feedback, and support via direct messaging as well as coping skill suggestions customized for each patient.

### In-Person IOP Treatment

Participants in this program received three 3-hour group sessions per week, 1 hour of individual or family therapy weekly, and one 30-minute appointment with a registered dietitian every other week (exactly the same as VIOP). Medical monitoring was conducted once per week at each onsite facility. Blind weighing was also used for this group. The program is comparable to VIOP, following the same group therapy schedule and treatment approaches. The exception was that the interpersonal process group occurred once per week and Recovery Record was not used to record meal intake during programming since onsite staff did so. It was, however, used throughout the week in the same manner as it was for the VIOP group. See Table S2 in Multimedia Appendix 1 for an example schedule of VIOP and in-person IOP treatment.

### Statistical Analyses

All analyses were performed in R software (version 4.4.3; The R Foundation) [32]. Descriptive analyses included calculating means, SDs, percentages, and the NPS (described above in Measures). Primary analyses involved logistic regressions. First, residualized change scores for all outcomes were created by saving residual variances after predicting discharge scores from admission scores, thus creating change scores free from associations with scores on admission. Multiple logistic regressions were then conducted predicting the program (reference group VIOP vs in-person IOP) from residualized change scores for each variable. Demographic and clinical features that varied significantly between VIOP and in-person IOP were included as covariates in multiple logistic regressions. Regression diagnostics, including Q-Q plots, residuals versus leverage plots, and residuals versus fitted values plots were performed to examine whether regression assumptions were met. This includes a normal distribution of residual values, despite the presence of skewness in some admission and discharge scores of variables, such as PHQ-9 item 9. Cohen *d* effect sizes for all effects irrespective of statistical significance are reported and interpreted as small (*d*≥0.3), medium (*d*≥0.5), and large (*d*≥0.8) [33]. Odds ratios and 95% CIs are presented for logistic regression results. Because our primary hypotheses were around the equivalence of VIOP compared to in-person IOP, no multiple test corrections were performed, as they are typically overly conservative, leading to increased chances of type II error (which would unduly bias our results in favor of our hypotheses rather than away from our hypotheses). In other words, by not using a multiple-test correction, we can be more confident that all nonsignificant findings (which are in line with our hypotheses) are more likely to be truly nonsignificant [34].

Paired-sample *t* tests were used to compare the admission and discharge scores of patients in VIOP and in-person IOP. Table S3 in Multimedia Appendix 1 presents independent-sample *t* tests used to compare admission, discharge, and change scores between patients across VIOP and in-person IOP using the Welch method, which is more robust to the possibility of unequal variances from largely unequal sample sizes than the more commonly used Student *t* test [35]. These results were also compared with bivariate linear regressions, which are also robust to unequal variances. Table S3 in Multimedia Appendix 1 is presented for the interpretability of raw differences and change scores but is considered secondary to the primary analyses described above.

A total of 4 sensitivity analyses were also conducted: (1) comparison of patients in VIOP and in-person IOP in the current sample with those only completing admission assessments; (2) comparison between VIOP patients in the early months of the study, who did not have an alternative in-person IOP option, and VIOP patients later in the study period who self-selected into VIOP when in-person IOP was also available; (3) comparison of VIOP and in-person IOP patients aged more than 18 years only; and (4) comparison of VIOP and in-person IOP patients aged 11-17 years.

### Ethical Considerations

This study was approved by the Salus Institutional Review Board (study title: Eating Recovery Center Outcome Research, protocol number ERC-001). Adult patients signed informed consent, while the parents of adolescent patients provided informed consent, and adolescents provided assent at admission and completed self-report questionnaires at admission and discharge. Study data are de-identified. Patients did not receive monetary compensation for completing questionnaires but were informed that benefits to participation may include an increased understanding of their personal condition due to assessment results being available to the patient and their care team before discharge. Patients were also informed their consent to use questionnaire responses in research would have no impact on their treatment.

## Results

Table 1 presents demographic and clinical information. Table 2 presents admission and discharge scores for the PHQ-9 and EDE-Q along with Cohen *d*, and NPS for VIOP and in-person IOP participants. Both groups were primarily female and White, with patients in VIOP (mean 29.03, range 12-63) presenting as somewhat older than in-person patients in IOP (mean 19.51, range 12-42). Other specified feeding or eating disorder (OSFED) was the most prevalent diagnosis for patients in VIOP (109/231, 47.2%), and in-person patients in IOP (17/39, 43.6%). Both programs resulted in significant improvements in depression and ED symptoms with large effect sizes, except for Item 9 of the PHQ-9, which improved with VIOP treatment with a small effect size and did not improve with in-person IOP treatment. NPS for VIOP treatment was 45.6, and NPS for in-person IOP treatment was 20.

Table S3 in Multimedia Appendix 1 compares raw values at admission, discharge, and change scores for VIOP versus in-person IOP. Patients in VIOP had significantly lower scores on the PHQ-9 total score, Item 9 of the PHQ-9, and the restraint subscale of the EDE-Q at discharge when compared to in-person patients with IOP. The patients with VIOP showed a greater improvement in depression scores than in-person patients with IOP. The patients with VIOP were significantly older (mean 28.03, SD 11.09; range 11-63) than in-person patients with IOP (mean 19.51, SD 6.98; range 12-42), had significantly different proportions of ED diagnoses, and had significantly more comorbid conditions (VIOP 1.23, SD 1.12; in-person IOP 0.33, SD 0.84).

Table 3 shows logistic regression results predicting the probability of a patient belonging to VIOP (vs in-person IOP) as a result of changes in each symptom, with age, diagnosis, and number of comorbid diagnoses as covariates (as these were significantly different between VIOP and in-person IOP). Table S4 in Multimedia Appendix 1 includes full model results. When controlling for age and diagnosis, patients were significantly more likely to have been discharged from VIOP (vs in-person IOP) if their PHQ-9 total score and item 9 of the PHQ-9 showed greater residualized change scores. In other words, after controlling for admission scores and relevant treatment group differences, patients in VIOP had greater decreases in PHQ-9 total score and item 9 than patients in in-person IOP.

**Figure 1:**
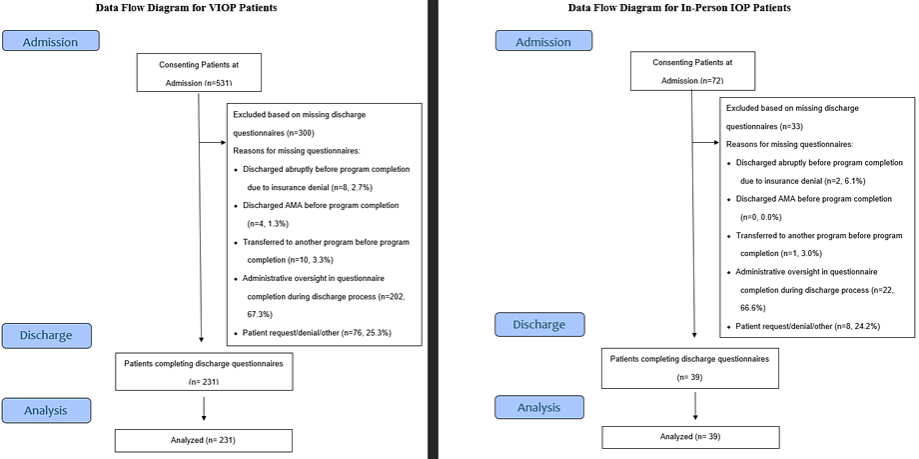
Flow diagram for patient recruitment.

Sensitivity analysis found no differences in diagnosis, comorbid diagnoses, age, gender identity, race, or clinical values at admission in VIOP or in-person patients with IOP in the current sample compared to VIOP or in-person patients with IOP with assessments at admission only. Additionally, all effect sizes of differences between VIOP or in-person patients with IOP in the current sample compared to those with assessments at admission only were small (all Cohen *d*<.01). Figure 1 data flow diagram shows the number and reasons for noncompletion for patients with assessments at admission only (see Table S5 in Multimedia Appendix 1 for associated STROBE [Strengthening the Reporting of Observational Studies in Epidemiology] checklist). Because the majority of patients missing discharge data were due to administration issues at discharge rather than patient-driven factors, and because of the lack of differences found, we believe patients are missing discharge questionnaires completely at random.

Additional sensitivity analyses found no differences in PHQ-9 or EDE-Q subscale and global scores at admission or admission-discharge changes between patients in VIOP in the early months of the study period who did not have an alternative in-person IOP option and patients in VIOP later in the study period who self-selected into VIOP when in-person IOP was also offered. Examining VIOP versus in-person patients in IOP aged more than 18 years only did not result in any changes to statistical significance or any meaningful changes to effect sizes across findings in PHQ-9 or EDE-Q subscale or global scores at admission or admission-discharge changes. Finally, examining VIOP versus in-person patients in IOP aged 11-17 years only did not result in any changes to statistical significance or any meaningful changes to effect sizes across findings in PHQ-9 or EDE-Q subscale or global scores at admission or admission-discharge changes.

**Table 1:** Virtual intensive outpatient program and in-person intensive outpatient demographic and clinical characteristics (N=270).

Variables		VIOP^a^ (n=231)		In-person IOP^b^ (n=39)	
		Admission	Discharge	Admission	Discharge
Age (years), mean (SD)		28.03 (11.09)	—^c^	19.51 (6.98)	—
**Gender identity, n (%)**					
	Female	196 (84.8)	—	35 (89.7)	—
	Male	10 (4.3)	—	2 (5.1)	—
	Nonbinary	12 (5.2)	—	0 (0)	—
	Declined to answer	13 (5.6)	—	2 (5.1)	—
**Race/ethnicity, n (%)**					
	White	175 (75.6)	—	30 (76.9)	—
	Black	8 (3.5)	—	1 (2.6)	—
	Asian	9 (3.9)	—	1 (2.6)	—
	Hispanic/Latinx	8 (3.5)	—	1 (2.6)	—
	Multiracial	7 (3)	—	3 (7.7)	—
	Declined to answer	24 (10.4)	—	3 (7.7)	—
**Diagnosis, n (%)**					
	AN-R^d^	39 (16.9)	—	15 (38.5)	—
	AN-BP^e^	16 (6.9)	—	2 (5.1)	—
	BN^f^	15 (6.5)	—	1 (2.6)	—
	BED^g^	45 (19.5)	—	3 (7.7)	—
	ARFID^h^	7 (3)	—	1 (2.6)	—
	OSFED^i^	109 (47.2)	—	17 (43.6)	—
**Comorbid diagnosis, n (%)**					
	Depressive disorder	116 (50.2)	—	5 (12.8)	—
	Anxiety disorder	126 (54.5)	—	6 (15.4)	—
	Substance use disorder	8 (3.5)	—	0 (0)	—
	Borderline personality disorder	6 (2.6)	—	0 (0)	—
	ADHD^j^	6 (2.6)	—	1 (2.6)	—
	Number of comorbid diagnoses, mean (SD)	1.23 (1.12)	—	0.33 (0.84)	—
	Length of stay (days), mean (SD)	—	58.84 (26.69)	—	57.33 (19.67)

^a^VIOP: virtual intensive outpatient program.

^b^IOP: intensive outpatient.

^c^Not applicable.

^d^AN-R: anorexia nervosa restricting type.

^e^AN-BP: anorexia nervosa. Binge/purge type.

^f^BN: bulimia nervosa.

^g^BED: binge eating disorder.

^h^ARFID: avoidant/restrictive food intake disorder.

^i^OSFED: other specified feeding or eating disorder.

^j^ADHD: attention-deficit hyperactivity disorder.

**Table 2:** Virtual intensive outpatient program and in-person intensive outpatient outcomes (N=270).

Variables	VIOP^a^ (n=231), mean (SD)					In-person IOP^b^ (n=39), mean (SD)			
	Admission	Discharge	*P* value	Cohen *d*	Admission		Discharge	*P* value	Cohen *d*
PHQ-9^c^	12.98 (5.87)	7.69 (5.33)	<.001	0.98	13.89 (6.58)		11.1 (6.24)	.003	0.52
PHQ-9 item 9	0.51 (0.8)	0.28 (0.61)	<.001	0.33	0.71 (0.87)		0.56 (0.88)	.38	0.15
EDEQ^d^ Eating concerns	2.97 (1.5)	1.41 (1.21)	<.001	1.07	2.79 (1.54)		1.47 (1.44)	<.001	1.02
EDEQ restraint	2.25 (1.7)	0.72 (1.04)	<.001	0.96	2.27 (1.84)		1.14 (1.31)	<.001	0.77
EDEQ Shape concern	4.44 (1.47)	2.96 (1.68)	<.001	0.99	4.19 (1.73)		2.89 (2.07)	<.001	0.94
EDEQ Weight concern	4.03 (1.55)	2.59 (1.63)	<.001	0.98	3.68 (1.75)		2.48 (2.02)	<.001	0.8
EDEQ Global	3.42 (1.33)	1.92 (1.23)	<.001	1.2	3.23 (1.51)		1.99 (1.58)	<.001	0.99
NPS^e^	—^f^	45.6	—	—	—		20	—	—

^a^VIOP: virtual intensive outpatient program.

^b^IOP: intensive outpatient.

^c^PHQ-9: Patient Health Questionnaire.

^d^EDEQ: Eating Disorder Examination Questionnaire.

^e^NPS: Net Promoter Score.

^f^Not applicable.

**Table 3:** Adjusted logistic regressions predicting virtual intensive outpatient program versus in-person intensive outpatient.

Residualized change score predictor^a^	b	SE	*t* test (*df*)	*P* value	OR^b^ (95% CI)
PHQ-9^c^	–0.14	0.05	–2.85 (263)	.004	0.87 (0.79-0.96)
PHQ-9 Item 9	–0.72	0.34	–2.13 (263)	.03	0.49 (0.25-0.93)
EDEQ^d^ Eating concerns	0.07	0.18	0.40 (263)	.69	1.08 (0.76-1.55)
EDEQ Restraint	–0.27	0.19	–1.48 (263)	.14	0.76 (0.53-1.1)
EDEQ Shape concerns	0.05	0.14	0.39 (263)	.69	1.06 (0.8-1.39)
EDEQ Weight concerns	0.05	0.14	0.35 (263)	.72	1.01 (0.71-1.43)
EDE-Q Global	0.01	0.18	0.04 (263)	.97	1.01 (0.71-1.43)

^a^Information is presented for each residualized change score coefficient predicting group assignment (VIOP vs in-person IOP), after also controlling for age, diagnosis, and number of comorbidities. Each row represents a separate regression model.

^b^OR: odds ratio.

^c^PHQ-9: Patient Health Questionnaire-9.

^d^EDEQ: Eating Disorder Examination Questionnaire.

## Discussion

### Overview

In a naturalistic comparison of VIOP versus in-person IOP services for EDs during the COVID-19 pandemic, patients in VIOP did not exhibit significantly different improvements in ED symptom scores. However, patients in VIOP did exhibit significantly greater improvements in depression scores and the PHQ-9 suicidal ideation item. Patients in VIOP were significantly older than in-person patients in IOP, had significantly different numbers of ED diagnoses, and significantly more comorbid psychiatric diagnoses. Together, these results suggest that VIOP treatment may improve treatment access in an equitable fashion for all patients, which has been a concern during the COVID-19 pandemic [36], without reducing treatment quality.

### Principal Findings

There were no significant differences in gender identity between treatment groups. When looking descriptively across each category, there were more nonbinary patients in VIOP. This could be due to nonbinary patients perhaps feeling more comfortable in a virtual environment rather than presenting in person for treatment, depending on numerous environmental factors [37], or it could simply be due to the greater number of patients in VIOP. Differences were generally found in the distribution of ED diagnoses and the number of comorbidities between treatment groups and were accounted for in analyses. Again, when descriptively looking across each category, however, it is unclear why patients with anorexia nervosa-restricting type might be more likely to seek in-person treatment than virtual treatment, and why patients with binge eating disorder would be more likely to seek virtual treatment. It is also unclear why patients with more comorbidities may be more likely to seek virtual treatment. Future studies may want to replicate these findings and include qualitative assessments of patients’ reasons for choosing one treatment setting over another.

Both treatment groups showed significant improvements in depression; however, the in-person IOP group did not improve on Item 9 of the PHQ-9, while the VIOP group did. This may be due to the smaller sample size of the in-person IOP group; larger numbers may have resulted in significant changes. Patient satisfaction was higher for the VIOP group, which can likely be attributed to the convenience of virtual treatment and reduced barriers to access.

### Comparison to Previous Work

This study’s findings extend evidence from the only 4 other studies examining outcomes in VIOP versus in-person IOP in ED treatment in 2 key ways [12]. Specifically, this study’s sample of VIOP patients is many times larger than the previous samples’, increasing confidence in the overall beneficial treatment effects of VIOP. In addition, this study’s VIOP and in-person IOP treatment time frames were largely overlapping due to the lifting of restrictions from in-person services during the COVID-19 pandemic, thus tempering the potential limitation for historical artifacts of different time periods to differentially affect outcomes in the 2 previous samples.

It has been estimated that only around one-quarter of individuals with EDs seek treatment for this disorder [38]. Many practical barriers to ED treatment have been identified, including limited access to treatment due to factors such as financial issues and insurance coverage, the time required to travel to the treatment facility, or lack of a nearby treatment facility. Personal barriers have also been identified, such as lack of recognition of the seriousness of the illness, as well as systemic barriers including shame and stigma surrounding EDs, and ethnic, racial, and cultural factors [39]. VIOP has the potential to negate some of these barriers, such as travel costs [40], and stigma associated with presenting in-person to a treatment facility. While it is beyond the scope of this study to investigate all these factors, age and race are 2 factors measured by this study where some initial descriptive trends may be examined for future work to investigate more deeply.

The descriptive trends of age in this study did not indicate that the disparities in treatment access by older age individuals, which already exist, were exacerbated by the telehealth modality of VIOP. Telehealth treatment can lead to increases in these disparities [5]; thus, it may be meaningful that patients with VIOP were older on average (with patients aged up to 63 years compared to 42 years in in-person IOP, and over 7%, 17/231 patients with VIOP aged more than 50 years). It may also be meaningful that in a higher level of care ED treatment, where the vast majority of patients are White, patients with VIOP had descriptively greater representation than in-person patients with IOP in every racial and ethnic minority category, aside from multiracial identity. It is crucial to acknowledge these are only descriptive observations, however. Future work should further investigate these trends in adequately powered samples. Similarly, future intervention development should go further by targeting telehealth interventions to reduce the larger disparities in treatment access.

### Strengths and Limitations

This study has several strengths worth acknowledging. First, it is the largest study to date of patients with ED receiving VIOP treatment. Second, the study was conducted in a naturalistic setting in which patients were presenting for a higher level of care treatment without recruitment and combined both patients who chose VIOP treatment over in-person options, as well as patients whose only option was VIOP treatment in lieu of in-person treatment, due to the pandemic. Both strengths are likely to increase the generalizability of these findings to VIOP treatment for EDs more broadly. Still, this study also has some limitations. First, the in-person IOP treatment sample was substantially smaller than the VIOP treatment sample. This is primarily due to the difficulty in collecting discharge assessments for in-person patients with IOP, as well as the more condensed time period in which in-person IOP was available due to COVID-19 restrictions. Nevertheless, there appears to be no meaningful difference in patients included in this sample from the broader IOP population. Second, age and diagnostic differences existed between VIOP and in-person IOP. Although these factors were controlled for in the analysis, future work should investigate the potential for demographic and clinical differences in these patient populations, define the mechanisms by which they arise, and any downstream impacts. This inclusion for future work also applies to other potentially relevant unmeasured factors, namely duration of illness. Third, when examining subgroups of individuals in sensitivity analyses, our cell sizes were smaller and may have impacted the nonsignificance of between-group findings. Finally, all outcomes were self-reported and occurred immediately after discharge. Thus, it is possible that observed outcomes were influenced by patient perception and that these improvements were not sustained. Future research should seek to measure a larger range of VIOP outcomes and measure VIOP outcomes at follow-up time points.

**Conclusions**

The COVID-19 pandemic required a dramatic shift in the way health care is delivered, including treatment for EDs. Results of telehealth treatment for EDs have been promising, and this study adds to this literature by demonstrating that ED patients in VIOP treatment did not have worse outcomes that ED patients in in-person IOP treatment. Although this study did not find disparities in access to VIOP treatment, future studies should still seek to target the digital divide question more directly with regard to ED telehealth treatment. Given the limited access in many areas to ED treatment of any kind, especially higher level of care treatment, practitioners and researchers alike should continue to seek both rapid expansion and rapid, rigorous evaluation of telehealth treatment for EDs.

## Appendix

Multimedia Appendix 1 Indications for virtual intensive outpatient/intensive outpatient treatment.

## Abbreviations

CALOCUS: Child and Adolescent Level of Care Utilization System
ED: eating disorder
EDE-Q: Eating Disorder Examination – Questionnaire
IOP: intensive outpatient
LOCUS: Level of Care Utilization System
NPS: Net Promoter Score
OSFED: other specified feeding or eating disorder
PHP: Partial Hospitalization Program
PHQ-9: Patient Health Questionnaire-9
STROBE: Strengthening the Reporting of Observational Studies in Epidemiology
VIOP: virtual intensive outpatient

## Data Availability

The datasets generated or analyzed during this study are available from the corresponding author on reasonable request.
