# Supplementary material for: Virtual Versus In-Person Intensive Outpatient Treatment for Eating Disorders During the COVID-19 Pandemic in United States–Based Treatment Facilities: Naturalistic Study
Source: J Med Internet Res. 2025 May 2;27:e66465. doi: 10.2196/66465 (PMC12084767; doi:10.2196/66465)
Supplement: Multimedia Appendix 1 [file jmir_v27i1e66465_app1.docx]

| Supplemental Table 1. Indications for VIOP/IOP Treatment | | | | | | | | |
| --- | --- | --- | --- | --- | --- | --- | --- | --- |
|  | Indications for IOP/VIOP vs. OP | Indications for IOP/VIOP vs. Higher Level of Care Treatment | | | | | | |
| Domain | Indication | Indication 1 | | Indication 2 | Indication 3 | | | Indication 4 |
| **Medical Comorbidity** | Medically stable to the extent that medical monitoring is not required by ERC medical teammates. | Medically stable to the extent that medical monitoring is not required by ERC medical teammates. | | Per Joint Commission standards, every patient will be seen within 2 weeks of admission by an outside medical provider for an eating disorder focused history and physical plus laboratory monitoring (usually CBC, CMP, Phosphorus, and Magnesium plus EKG and/or thyroid functioning, if indicated). Patient must be appropriate for minimal lab monitoring during IOP care. No ERC physician or nursing monitoring is provided. | Minimal refeeding risk with no recent significant pattern of weight loss. Stable, normal labs expected especially phosphorus, sodium, potassium, & CO2 levels. | | | Vital signs: normal resting heart rate (generally greater than 60), normal EKG. No symptoms of dizziness, exercise intolerance or excessive fatigue and no need for monitoring of orthostatic vital signs. |
| **Weight (% of NBW)** | Generally >80% (as opposed to >85% for OP). | If weight restoration indicated, generally less than 5-10% NBW or IBW to restore; recommended rate of weight restoration: 1.0 lbs. per week. | | | | | | |
| **Structure Needed for Eating and to Restore Weight** | Self-sufficient at both IOP and OP. | 3 meals per week. Note: virtual IOP patient must have access to quiet private area where a meal can be consumed and which has computer/internet connection > 3 hours a day, 3 days a week. | | | *Additional indication for children and adolescents:* Compliant with parent, legal guardian or team approved adult direction. Parent, legal guardian, or team approved adult must be able to have meal with patient. | | | |
| **Ability to Control Compulsive Exercise** | Some degree of external structure beyond self-control required (as opposed to self-control only for OP) | Motivated to change behaviors with exercise, sport or activity that moves away from a compulsive mindset with the need for some external structure and support. Activity is not impacting quality of life, physical health, and/or emotional health to the exclusion of work, relationships, and recreation. | | *Indication for IOP and Virtual IOP:* Patient readily shares this information with their team and/or adult support persons. | *Indication for IOP and Virtual IOP:* Patient is compliant with redirection to reduce excessive movement. | | | *Indication for IOP:* Patient compliant with activity monitor when recommended by team. |
| **Symptom Intensity and Frequency (e.g., food restriction, binging behaviors, and purging behaviors including laxatives and diuretics)** | Able to reduce incidents of purging in an unstructured setting, no significant medical complications, or other abnormalities suggesting need for hospitalization (for both OP and IOP) | No replacement/ supplemental nutrition for missed portions of meal plan is provided. Symptom frequency moderate: 4-7 times per week, possibly fewer, depending on individual symptom presentation. | | *Indication for IOP:* Some degree of external structure beyond self-control or support system is required to prevent symptom use. Can ask for and use support from others or use cognitive and behavioral skills to inhibit purging. | *Additional Indication for Virtual IOP:* ED symptoms are decreasing in intensity, frequency, and duration. Patient is compliant with recommendations for medical monitoring by outpatient providers when ERC team recommends. | | | *Additional indication for children and adolescents:*  Compliance with direction from parents or caregivers. |
| **Motivation to Recover, Including Cooperativeness, Insight, and Ability to Control Obsessive Thoughts** | Fair motivation acceptable (as opposed to fair-to-good motivation for OP) | *Indication for IOP:* Reasonable acceptance or knowledge of reality of the illness and takes reasonable responsibility for recovery. Some motivation to change and participate in programming exists. Some confidence in ability to change/acceptance of responsibility. | | *Additional indication for children and adolescents:* Generally compliant with parent direction and honest in communication within family system. | | *Additional Indication for Virtual IOP:* Same as onsite IOP plus patient has the ability to access internal support and support system to log on as scheduled, participate in safety planning and treatment goal setting and communicate to team and family support when more support is needed. | | |
| **Co-Occurring Disorders (e.g., substance use, depression, and anxiety)** | Presence of comorbid condition may influence level of care for OP vs. IOP, or higher | Co-occurring psychiatric or substance use symptoms impact functioning but are adequately stabilized and do not require on site psychiatric evaluation and management. Patient agrees to abstain from all substance use during treatment course. Patient agrees to see their outpatient team, including an outpatient psychiatrist, during IOP treatment course and to follow treatment recommendations. Patient agrees to allow IOP team communication and care coordination with entire outpatient treatment team. Urine toxicology screening may be required, ordered by the Outpatient team for accountability and progress in treatment. | | | | | | |
| **Environmental Stress** | Other able to provide adequate emotional and practical support and structure (for both IOP and OP). | *Indication for Virtual IOP:* Home environment is not destabilizing or a perpetuating factor for psychiatric symptoms. | | *Additional indication for children and adolescents:* Parent, legal guardian or a team approved alternative adult to present to manage safety concerns if they arise during online treatment. Parent, legal guardian, or a team approved alternative adult is present to support virtual meal groups. | *Additional indication for children and adolescents:* Parent, legal guardian, or a team approved alternative adult is able to provide support and structure. | | | *Additional indication for children and adolescents:* Parent, legal guardian, or a team approved alternative adult is compliant with attending parent/caregive skills groups. |
| **Geographic Availability of a Treatment Program - Onsite** | Patient lives near treatment setting (for both IOP and OP) | Patient lives near a treatment center. | | | Significant and consistent at home support is available, and in a safe living environment. | | | |
| **Geographic Availability of a Treatment Program - Virtual** | Patient does not live near onsite treatment setting or has other problems with access to onsite care. (for both IOP and OP) | Patient does not live near onsite treatment setting or has other problems with access to onsite care. | | Patient can consistently attend programming in a state in which ERC is licensed. | Patient can access an internet connection that supports streaming. | | | Significant and consistent at home support is available, and in a safe living environment |
| **Geographic Availability of a Treatment Program – Virtual for child/adolescent** | Same as Virtual, plus: | Significant at-home support consistently available. | | Parent, legal guardian, or a team approved alternative adult is present to support virtual meal groups. | Patient moderately compliant to very compliant with parent, legal guardian or team approved alternative adult direction/support. | | | |
| **Suicidality** | If suicidality is present, monitoring may be needed depending on level of risk. | Some history of risk and/or distress, but there is no current suicidal ideation, plan, or intent. Not a harm to others. IOP/VIOP patients are expected to be readily forthcoming to team and/or family and willing to accept support with incidents of self-harm or changes on suicidality. | | Fleeting thoughts but no plan/intent. Does not persist on most days. | | | Has no active plan, means, or intention. Ideation presents more like intrusive thinking. | |
| **Self-injuriousness** | If self-injury is present, monitoring may be needed depending on level of risk. | Some history of risk and/or distress, but there is no current suicidal ideation, plan, or intent. Not a harm to others. IOP/VIOP patients are expected to be readily forthcoming to team and/or family and willing to accept support with incidents of self-harm or changes on suicidality. | | If present, medical intervention not necessary. Self-inflicted wounds are limited to scratches or abrasions, hair pulling, hitting self, or otherwise causing harm. | | Shows an ability to report, intervene, and use coping skills a majority of the time. Ability to ground self fairly consistently and apply coping skills training. Ability to self-regulate mood/anxiety fairly consistently. | | |
| **Suicidality and/or Self-harm – Child/Adolescent** | Same as Suicidality and Self-Injuriousness, plus: | | Demonstrates consistent ability and willingness to share thoughts, urges, and behaviors with parent/caregiver & accept support. | | | | | |
| **Other expectations for IOP:** | Same additional expectations for IOP and OP. | Medication compliant and safe to have supply of medications at home. | | Mood stability is fairly consistent: no destabilizing manic or hypomanic symptoms, no psychosis. | | | | |
| *Note.* Many indications adapted from Table 8 of APA Practice Guideline for the Treatment of Patients with Eating Disorders, Third Edition | | | | | | | | |

*Supplemental Table 2.* In-Person IOP and VIOP Example Schedules.

| Time | Monday | Wednesday | Thursday |
| --- | --- | --- | --- |
| 5:00pm - 6:00pm | Didactic Skills Group Led by Therapists | Didactic Skills Group Led by Therapists | Didactic Skills Group Led by Therapists |
| 6:00pm - 7:00pm | Monitored and Supported Dinner | Monitored and Supported Dinner | Monitored and Supported Dinner |
| 7:00 pm - 8:00pm | Didactic Skills Group Led by Therapists | Process Group Supported by Therapists | Didactic Skills Group Led by Therapists |

| *Supplemental Table 3*. Comparing VIOP and IOP Characteristics and Outcomes Raw Scores | | | | | | | | | |
| --- | --- | --- | --- | --- | --- | --- | --- | --- | --- |
|  | Difference on Admission | p | Cohen’s d | Difference on Discharge | p | Cohen’s d | Difference in Change | p | Cohen’s d |
| Age (M, SD) | **8.52 (9.27)** | **<.001** | **0.92** | - | - | - | - | - | - |
| Gender Identity (N, %) | - | .34^†^ | - | - | - | - | - | - | - |
| Race/Ethnicity (N, %) | - | .70^†^ | - | - | - | - | - | - | - |
| Diagnosis (N, %) | - | **<.001** | - | - | - | - | - | - | - |
| # Comorbid Diagnoses (M, SD) | 0.90 | **.02** | **0.4** |  |  |  |  |  |  |
| Length of Stay, Days (M, SD) | - | - | - | 1.51 (23.45) | .74 | 0.06 | - | - | - |
| PHQ-9 (M, SD) | -0.91 (6.23) | .38 | -0.15 | **-3.42 (5.80)** | **<.001** | **-0.59** | **-2.67 (5.26)** | **.005** | **-0.51** |
| PHQ-9 Item 9 (M, SD) | -0.20 (0.83) | .16 | -0.24 | **-0.29 (0.76)** | **.01** | **-0.38** | -0.10 (0.81) | .42 | -0.13 |
| EDEQ Eating Concerns (M, SD) | 0.18 (1.52) | .49 | 0.12 | -0.06 (1.33) | .78 | -0.04 | -0.24 (1.38) | .34 | -0.17 |
| EDEQ Restraint (M, SD) | -0.02 (1.77) | .94 | -0.01 | **-0.42 (1.18)** | **.03** | **-0.36** | -0.40 (1.54) | .15 | -0.26 |
| EDEQ Shape Concerns (M, SD) | 0.25 (1.60) | .34 | 0.16 | 0.08 (1.88) | .80 | 0.04 | -0.17 (1.45) | .50 | -0.12 |
| EDEQ Weight Concerns (M, SD) | 0.34 (1.65) | .21 | 0.21 | 0.11 (1.83) | .72 | 0.06 | -0.24 (1.48) | .35 | -0.16 |
| EDEQ Global (M, SD) | 0.19 (1.42) | .42 | 0.13 | -0.07 (1.42) | .74 | -0.05 | -0.26 (1.25) | .23 | -0.21 |
| *Note.* M=Mean. ^†^Chi-Square Test of Independence. Negative values indicate VIOP score is lower than IOP score. For negative value change scores, VIOP score decreased more than IOP score. PHQ-9=Patient Health Questionnaire; EDEQ=Eating Disorder Examination Questionnaire. | | | | | | | | | |

Supplemental Table 4. Full Model Results of Adjusted Logistic Regressions Predicting VIOP Versus in-Person IOP.

|  | | | | | |
| --- | --- | --- | --- | --- | --- |
| *Table 3.* Adjusted Logistic Regressions Predicting VIOP Versus in-Person IOP | | | | | |
| Model & Predictors | b | SE | t | p-value | OR [95% C.I.] |
| **Residualized PHQ-9 Change Predicting VIOP Versus In-Person IOP (Ref=VIOP)** | | | | | |
| Intercept | 1.73 | 1.45 | 1.2- | 0.23 | 5.68 [0.39, 165] |
| PHQ-9 Change | **-0.14** | **0.05** | **-2.85** | **.004** | **0.87 [0.79, 0.96]** |
| Admission PHQ-9 | 0.02 | 0.04 | 0.36 | .72 | 1.02 [0.93, 1.11] |
| Age | **0.12** | **0.04** | **3.01** | **.003** | **1.13 [1.05, 1.23]** |
| AN-R Diagnosis | **-2.63** | **1.15** | **-2.30** | **.02** | **0.07 [<0.01, 0.50]** |
| ARFID Diagnosis | -1.70 | 1.60 | -1.06 | .29 | 0.18 [<0.01, 5.90] |
| BED Diagnosis | -1.56 | 1.28 | -1.22 | .22 | 0.21 [0.01, 2.11] |
| BN Diagnosis | 12.49 | 928.24 | 0.01 | .99 | 265995 [<0.01, 1.04e^139^] |
| OSFED Diagnosis | -1.95 | 1.13 | -1.73 | .08 | 0.14 [0.01, 0.91] |
| # of Comorbidities | **0.39** | **0.19** | **1.99** | **.047** | **1.47 [1.02, 2.20]** |
| **Residualized PHQ-9 Item 9 Change Predicting VIOP Versus In-Person IOP (Ref=VIOP)** | | | | | |
| Intercept | 0.49 | 1.35 | 0.37 | .71 | 1.63 [0.14, 41.63] |
| PHQ-9 Item 9 Change | **-0.72** | **0.34** | **-2.13** | **.03** | **0.49 [0.25, 0.93]** |
| Admission PHQ-9 Item 9 | 0.02 | 0.27 | 0.09 | .93 | 1.02 [0.62, 1.79] |
| Age | **0.13** | **0.04** | **3.27** | **.001** | **1.14 [1.06, 1.24]** |
| AN-R Diagnosis | **-2.25** | **1.12** | **-2.02** | **.04** | **0.10 [0.01, 0.65]** |
| ARFID Diagnosis | -1.70 | 1.56 | -1.09 | .28 | 0.18 [0.01, 5.63] |
| BED Diagnosis | -1.43 | 1.25 | -1.15 | .25 | 0.24 [0.01, 2.25] |
| BN Diagnosis | 12.90 | 952.07 | 0.01 | .99 | 399262 [<0.01, 6.98e^133^] |
| OSFED Diagnosis | -1.62 | 1.10 | -1.47 | .14 | 0.20 [0.01, 1.19] |
| # of Comorbidities | **0.39** | **0.20** | **1.98** | **.048** | **1.48 [1.02, 2.23]** |
| **Residualized EDEQ Eating Concerns Change Predicting VIOP Versus In-Person IOP (Ref=VIOP)** | | | | | |
| Intercept | -0.44 | 1.16 | -0.39 | .70 | 0.64 [0.07, 7.21] |
| EDEQ Eating Concerns Change | 0.07 | 0.18 | 0.40 | .69 | 1.08 [0.76, 1.55] |
| Admission EDEQ Eating Concerns | -0.17 | 0.17 | -1.01 | .31 | 0.84 [0.60, 1.17] |
| Age | **0.14** | **0.04** | **3.43** | **<.001** | **1.15 [1.07, 1.26]** |
| AN-R Diagnosis | -1.40 | 0.84 | -1.66 | .10 | 0.25 [0.03, 1.10] |
| ARFID Diagnosis | -0.85 | 1.40 | -0.60 | .55 | 0.43 [0.03, 11.42] |
| BED Diagnosis | -0.53 | 1.02 | -0.52 | .60 | 0.59 [0.07, 4.31] |
| BN Diagnosis | 14.15 | 940.63 | 0.02 | .99 | 391226 [<0.01, 5.08e^132^] |
| OSFED Diagnosis | -0.63 | 0.82 | -0.77 | .44 | 0.53 [0.08, 2.23] |
| # of Comorbidities | 0.30 | 0.19 | 1.57 | .12 | 1.35 [0.94, 2.00] |
| **Residualized EDEQ Restraint Change Predicting VIOP Versus In-Person IOP (Ref=VIOP)** | | | | | |
| Intercept | -0.63 | 1.13 | -0.56 | .58 | 0.53 [0.06, 5.64] |
| EDEQ Restraint Change | -0.27 | 0.19 | -1.48 | .14 | 0.76 [0.53, 1.10] |
| Admission EDEQ Restraint | 0.08 | 0.13 | 0.64 | .52 | 1.09 [0.84, 1.42] |
| Age | **0.13** | **0.04** | **3.37** | **<.001** | **1.14 [1.07, 1.24]** |
| AN-R Diagnosis | -1.28 | 0.85 | -1.51 | .13 | 0.28 [0.04, 1.24] |
| ARFID Diagnosis | -0.65 | 1.40 | -0.47 | .64 | 0.52 [0.03, 13.77] |
| BED Diagnosis | -0.80 | 1.06 | -0.76 | .45 | 0.45 [0.05, 3.48] |
| BN Diagnosis | 14.01 | 935.68 | 0.02 | .99 | 215442 [<0.01, 9.70e^141^] |
| OSFED Diagnosis | -0.71 | 0.84 | -0.84 | .40 | 0.49 [0.07, 2.16] |
| # of Comorbidities | 0.30 | 0.19 | 1.55 | .12 | 1.35 [0.94, 2.00] |
| **Residualized EDEQ Shape Concerns Change Predicting VIOP Versus In-Person IOP (Ref=VIOP)** | | | | | |
| Intercept | -0.61 | 1.21 | -0.50 | .62 | 0.55 [0.05, 6.71] |
| EDEQ Shape Concerns Change | 0.05 | 0.14 | 0.39 | .69 | 1.06 [0.80, 1.39] |
| Admission EDEQ Shape Concerns | -0.10 | 0.17 | -0.58 | .56 | 0.91 [0.65, 1.26] |
| Age | **0.14** | **0.04** | **3.43** | **<.001** | **1.15 [1.07, 1.25]** |
| AN-R Diagnosis | -1.29 | 0.83 | -1.54 | .12 | 0.28 [0.04, 1.20] |
| ARFID Diagnosis | -0.68 | 1.43 | -0.48 | .63 | 0.51 [0.03, 14.02] |
| BED Diagnosis | -0.59 | 1.02 | -0.58 | .56 | 0.55 [0.06, 4.05] |
| BN Diagnosis | 14.18 | 938.46 | 0.02 | .99 | 440198 [<0.01, 1.05e^152^] |
| OSFED Diagnosis | -0.63 | .82 | -0.76 | .45 | 0.53 [0.08, 2.25] |
| # of Comorbidities | 0.31 | 0.19 | 1.62 | .10 | 1.37 [0.95, 2.03] |
| **Residualized EDEQ Weight Concerns Change Predicting VIOP Versus In-Person IOP (Ref=VIOP)** | | | | | |
| Intercept | -0.67 | 1.17 | -0.58 | .57 | **0.51 [0.05, 5.77]** |
| EDEQ Weight Concerns Change | 0.05 | 0.14 | 0.35 | .72 | 1.01 [0.71, 1.43] |
| Admission EDEQ Weight Concerns | -0.08 | 0.16 | -0.47 | .64 | **0.93 [0.67, 1.27]** |
| Age | **0.14** | **0.04** | **3.42** | **<.001** | **1.15 [1.07, 1.25]** |
| AN-R Diagnosis | -1.29 | 0.84 | -1.54 | .12 | 0.28 [0.04, 1.21] |
| ARFID Diagnosis | -0.64 | 1.41 | -0.45 | .65 | 0.53 [0.03, 14.24] |
| BED Diagnosis | -0.62 | 1.02 | -0.60 | .55 | 0.54 [0.06, 3.96] |
| BN Diagnosis | 14.14 | 940.55 | 0.02 | .99 | 386457 [<0.01, 1.72e^138^] |
| OSFED Diagnosis | -0.62 | 0.82 | -0.76 | .45 | 0.54 [0.08, 2.26] |
| # of Comorbidities | 0.31 | 0.19 | 1.61 | .11 | 1.36 [0.95, 2.02] |
| **Residualized EDEQ Global Change Predicting VIOP Versus In-Person IOP (Ref=VIOP)** | | | | | |
| Intercept | -0.56 | 1.18 | -0.47 | .64 | 0.57 [0.06, 6.69] |
| EDE-Q Global Change | 0.01 | 0.18 | 0.04 | .97 | 1.01 [0.71, 1.43] |
| Admission EDEQ Global | -0.06 | 0.18 | -0.36 | .72 | 0.94 [0.66, 1.33] |
| Age | **0.14** | **0.04** | **3.41** | **<.001** | **1.15 [1.07, 1.25]** |
| AN-R Diagnosis | -1.31 | 0.84 | -1.56 | .12 | 0.27 [0.04, 1.19] |
| ARFID Diagnosis | -0.72 | 1.42 | -0.50 | .62 | 0.49 [0.03, 13.33] |
| BED Diagnosis | -0.63 | 1.02 | -0.62 | .53 | 0.53 [0.06, 3.87] |
| BN Diagnosis | 14.07 | 946.00 | 0.02 | .99 | 294162 [<0.01, 1.78e^137^] |
| OSFED Diagnosis | -0.65 | 0.82 | -0.79 | .43 | 0.52 [0.08, 2.21] |
| # of Comorbidities | 0.31 | 0.19 | 1.62 | .11 | 1.36 [0.95, 2.02] |
| *Note.* Information is presented for each residualized change score coefficient predicting group assignment (VIOP vs. in-person IOP), after also controlling for age, diagnosis, and number of comorbidities. Each separate regression model includes all predictors and the intercept, and the primary residualized change score noted in Table 3 is the predictor in each model labeled “Change”. For diagnosis categorical variable, reference group=AN-BP. e^xxx^=Upper limites of 95% CI for Odds Ratios of OSFED predictor are expontential, likely due to very small cell sizes creating poor estimates. PHQ-9=Patient Health Questionnaire; EDEQ=Eating Disorder Examination Questionnaire. AN-R=Anorexia Nervosa – Restricting Type; AN-BP=Anorexia Nervosa – Binge/Purge Type; BN=Bulimia Nervosa; BED=Binge Eating Disorder; ARFID=Avoidant/Restrictive Food Intake Disorder; OSFED=Other Specified Feeding or Eating Disorder. | | | | | |

Supplemental Table 5. Strobe Checklist

STROBE Statement—Checklist of items that should be included in reports of ***cohort studies***

|  | Item No | Recommendation | Page No |
| --- | --- | --- | --- |
| **Title and abstract** | 1 | (*a*) Indicate the study’s design with a commonly used term in the title or the abstract | 1 |
|  |  | (*b*) Provide in the abstract an informative and balanced summary of what was done and what was found | 3 |
| Introduction | | | |
| Background/rationale | 2 | Explain the scientific background and rationale for the investigation being reported | 5-6 |
| Objectives | 3 | State specific objectives, including any prespecified hypotheses | 6 |
| Methods | | | |
| Study design | 4 | Present key elements of study design early in the paper | 7-13 |
| Setting | 5 | Describe the setting, locations, and relevant dates, including periods of recruitment, exposure, follow-up, and data collection | 7-11 |
| Participants | 6 | (*a*) Give the eligibility criteria, and the sources and methods of selection of participants. Describe methods of follow-up | 7-11 |
|  |  | (*b*) For matched studies, give matching criteria and number of exposed and unexposed | N/A |
| Variables | 7 | Clearly define all outcomes, exposures, predictors, potential confounders, and effect modifiers. Give diagnostic criteria, if applicable | 8-13 |
| Data sources/ measurement | 8* | For each variable of interest, give sources of data and details of methods of assessment (measurement). Describe comparability of assessment methods if there is more than one group | 7-11 |
| Bias | 9 | Describe any efforts to address potential sources of bias | 11-13 |
| Study size | 10 | Explain how the study size was arrived at | 7, 17-18 |
| Quantitative variables | 11 | Explain how quantitative variables were handled in the analyses. If applicable, describe which groupings were chosen and why | 12-13 |
| Statistical methods | 12 | (*a*) Describe all statistical methods, including those used to control for confounding | 12-13 |
|  |  | (*b*) Describe any methods used to examine subgroups and interactions | 12-13 |
|  |  | (*c*) Explain how missing data were addressed | 7, 12-13 |
|  |  | (*d*) If applicable, explain how loss to follow-up was addressed | 7, 12-13 |
|  |  | (*e*) Describe any sensitivity analyses | 12-13 |
| Results | | |  |
| Participants | 13* | (a) Report numbers of individuals at each stage of study—eg numbers potentially eligible, examined for eligibility, confirmed eligible, included in the study, completing follow-up, and analysed | 7, 17-18 |
|  |  | (b) Give reasons for non-participation at each stage | 7, 17-18 |
|  |  | (c) Consider use of a flow diagram | 17-18 |
| Descriptive data | 14* | (a) Give characteristics of study participants (eg demographic, clinical, social) and information on exposures and potential confounders | 13-15 |
|  |  | (b) Indicate number of participants with missing data for each variable of interest | 13-15 |
|  |  | (c) Summarise follow-up time (eg, average and total amount) | 7, 13-15 |
| Outcome data | 15* | Report numbers of outcome events or summary measures over time | 13-18 |

| Main results | 16 | (*a*) Give unadjusted estimates and, if applicable, confounder-adjusted estimates and their precision (eg, 95% confidence interval). Make clear which confounders were adjusted for and why they were included | 13-16, 33 |
| --- | --- | --- | --- |
|  |  | (*b*) Report category boundaries when continuous variables were categorized | N/A |
|  |  | (*c*) If relevant, consider translating estimates of relative risk into absolute risk for a meaningful time period | N/A |
| Other analyses | 17 | Report other analyses done—eg analyses of subgroups and interactions, and sensitivity analyses | 16, 33 |
| Discussion | | | |
| Key results | 18 | Summarise key results with reference to study objectives | 19 |
| Limitations | 19 | Discuss limitations of the study, taking into account sources of potential bias or imprecision. Discuss both direction and magnitude of any potential bias | 20, 22 |
| Interpretation | 20 | Give a cautious overall interpretation of results considering objectives, limitations, multiplicity of analyses, results from similar studies, and other relevant evidence | 19-22 |
| Generalisability | 21 | Discuss the generalisability (external validity) of the study results | 19-22 |
| Other information | | | |
| Funding | 22 | Give the source of funding and the role of the funders for the present study and, if applicable, for the original study on which the present article is based | N/A |

*Give information separately for exposed and unexposed groups.

**Note:** An Explanation and Elaboration article discusses each checklist item and gives methodological background and published examples of transparent reporting. The STROBE checklist is best used in conjunction with this article (freely available on the Web sites of PLoS Medicine at http://www.plosmedicine.org/, Annals of Internal Medicine at http://www.annals.org/, and Epidemiology at http://www.epidem.com/). Information on the STROBE Initiative is available at http://www.strobe-statement.org.
